# SHP2在吸烟肺癌患者肿瘤组织中的表达及意义

**DOI:** 10.3779/j.issn.1009-3419.2010.09.08

**Published:** 2010-09-20

**Authors:** 雪梅 战, 红岩 董, 崇伟 孙, 丽丽 刘, 丹 王, 志永 韦

**Affiliations:** 276000 临沂，山东省临沂市人民医院病理科 Department of Pathology, Linyi People's Hospital of Shandong Province, Linyi 276003, China

**Keywords:** 肺肿瘤, 吸烟, 蛋白酪氨酸磷酸酶SHP2, 免疫组织化学, 荧光原位杂交, Lung neoplasms, Smoking, Protein tyrosine phosphatase SHP2, Immunohistochemistry, Fluorescence in situ hybridization

## Abstract

**背景与目的:**

蛋白质的磷酸化和去磷酸化是肺癌发生的重要机制，而吸烟是导致肺癌发生发展的重要危险因素，本研究旨在探讨蛋白酪氨酸磷酸酶SHP2在非小细胞肺癌(non-small cell lung cancer, NSCLC)和小细胞肺癌(small cell lung cancer, SCLC)肿瘤组织中的表达及其临床意义，并初步探讨不同的吸烟指数与SHP2表达水平的关系及可能机制。

**方法:**

采用免疫组织化学技术(Invision法)和荧光原位杂交技术(FISH法)检测53例肺癌组织中SHP2的表达和SHP2 mRNA的扩增情况。

**结果:**

SHP2在15例正常支气管上皮内弱阳性率为80%(亦为总阳性率)；在48例NSCLC中弱阳性率为35.4%，中度阳性率为43.8%，强阳性率为6.2%(总阳性率为85.4%)；在5例SCLC中弱阳性率为0%，中度阳性率为80%，强阳性率为20%(总阳性率为100%)。SHP2在27例非吸烟NSCLC患者肿瘤组织中的弱阳性率为40.7%，中度阳性率为37.4%，强阳性率为3.7%(总阳性率为81.5%)；SHP2在吸烟指数≥400的21例NSCLC患者肿瘤组织中弱阳性率为23.8%，中度阳性率为71.4%，强阳性率为4.7%(总阳性率为100%)。等级计数资料秩和检验结果表明，肺癌组织中SHP2表达的阳性率显著高于正常支气管上皮(*P*＜0.05)，SCLC中SHP2的阳性率高于NSCLC(*P*＜0.05)，吸烟指数≥400的NSCLC癌组织中SHP2阳性率高于非吸烟患者(*P*＜0.05)。

**结论:**

吸烟NSCLC患者肿瘤组织中SHP2的高表达可能与吸烟相关；SHP2可能在肺癌发生发展中起一定的作用；SHP2可能为肺癌治疗的药物研发提供新思路。

## Abstract (en)

**Background and objective:**

It has been proved that protein phosphorylation and dephosphorylation were important mechanisms in lung cancer development, and tobacco smoking is an important risk factor of lung cancer. The aim of this study is to investigate the expression and clinical significance of protein tyrosine phosphatase SHP2 in non-small cell lung cancer (NSCLC) and small cell lung cancer (SCLC); the relationship between tobacco smoking and the expression of SHP2 is also studied.

**Methods:**

Immunohistochemistry (Invision) and fluorescence in situ hybridization (FISH) were used to detect the expression of SHP2 and the augment of SHP2 mRNA in the 53 lung cancer specimens.

**Results:**

The weak positive rate of SHP2 was 80% (which was also the total positive rate) in normal bronchial epithelium. The weak, moderate and strong positive rates were 35.4%, 43.8% and 6.2% (total positive rate was 85.4%) in 48 NSCLC patients, 0%, 80% and 20% (total positve rate was 100%) in 5 SCLC patients, 40.7%, 37.4% and 3.7% (total positive rate was 81.5%) in the tumor tissues of 27 NSCLC patients who didn't smoke and 23.8%, 71.4% and 4.7% (total positive rate was 100%) in the tumor tissues of 21 NSCLC patients whose smoking indexes were ≥400. Significant differences of SHP2 expression were observed between tumor tissues and normal bronchial epithelium, NSCLC and SCLC, and between different smoking indexes (*P* < 0.05).

**Conclusion:**

The enhancement of SHP2 expression in the tumor tissues of NSCLC patients who smoke may be correlated with tobacco smoking; SHP2 may play certain role in the development of lung cancer; SHP2 prospectively provides new ideas for the drug research and development of lung cancer treatment.

肺癌分为非小细胞肺癌(non-small cell lung cancer, NSCLC)和小细胞肺癌(small cell lung cancer, SCLC)，是影响人类生存的重大疾病之一，全世界每年死于肺癌的人数占恶性肿瘤死亡总数的17.8%^[1]^。SHP2是含有SH2结构域的蛋白酪氨酸磷酸酶，在细胞生长、生存、侵袭、迁移等生物过程中具有重要的作用，其基因突变可导致多种恶性肿瘤的发生^[2]^，国内外有关SHP2在肺癌组织中表达情况的报道较少。吸烟是引起肺癌的主要危险因素，每年由于吸烟导致肺癌及其它肿瘤的死亡人数达100万。研究^[3]^表明，吸烟可导致多种原癌基因突变，继而引起细胞持续性增殖和癌变，有关SHP2在吸烟肺癌患者癌组织中的表达情况目前尚未见报道。本研究采用免疫组化和荧光原位杂交技术探讨SHP2在吸烟肺癌患者肿瘤组织中的蛋白表达及mRNA扩增情况，现报道如下。

## 1 材料与方法

### 1.1 病例资料

收集山东省临沂市人民医院2008年8月-2009年8月手术切除肺癌病例53例，平均年龄58.1岁(30岁-77岁)，男性32例，女性21例；NSCLC 48例(鳞癌17例，腺癌19例，细支气管肺泡癌8例，其它类型4例)，SCLC 5例，选择15例癌组织旁正常支气管上皮作为空白对照。其中，非吸烟患者为27例，吸烟指数≥ 400者为21例(均为NSCLC，5例SCLC中有4例吸烟史不详)。

### 1.2 石蜡包埋组织免疫组化检测

SHP2单克隆抗体购自美国Epitomics公司，采用Invision法，分为实验组、空白对照组(加PBS代替抗体)和阳性对照组(乳腺癌组织)。SHP2蛋白定位于细胞质和细胞核，阳性产物为棕黄色颗粒。阳性细胞＜6%为0分，6%-25%为1分，26%-50%为2分，51%-75%为3分，＞75%为4分；阳性强度：黄色为1分，棕黄色为2分，棕褐色为3分；两者积分相乘，0分为阴性(-)，1分-4分为弱阳性(+)，5分-8分为中度阳性(++)，9分-12分为强阳性(+++) ^[4]^。

### 1.3 石蜡包埋组织荧光原位杂交(FISH)检测

人SHP2原位杂交检测试剂盒购自武汉博士德生物工程有限公司，探针针对石蜡包埋组织内的SHP2 mRNA，探针序列为5’-AGTATTACATGGAACATCACGGGCAATTAAAAGA G-3’、5’-ACAAGGGAATACGGAGAGAACGGTCTGGCA ATACC-3’及5’-ATGAGAGAAGACAGTGCTAGAGTCTATG AAAACGT-3’，按照说明书进行操作。探针与胞质内靶基因SHP2 mRNA结合，经FITC标记，阳性者在荧光显微镜下胞质呈亮绿色。实验分为实验组、空白对照组(加预杂交液代替探针)和阳性对照组(乳腺癌组织)。

### 1.4 统计学分析

采用SPSS软件进行等级计数资料的秩和检验，以*P*＜0.05为差异具有统计学意义。

## 2 结果

### 2.1 免疫组化检测结果

SHP2在正常的支气管上皮内呈弱阳性，而在多例肺癌组织中呈广泛强阳性(图 1)。

**1:**
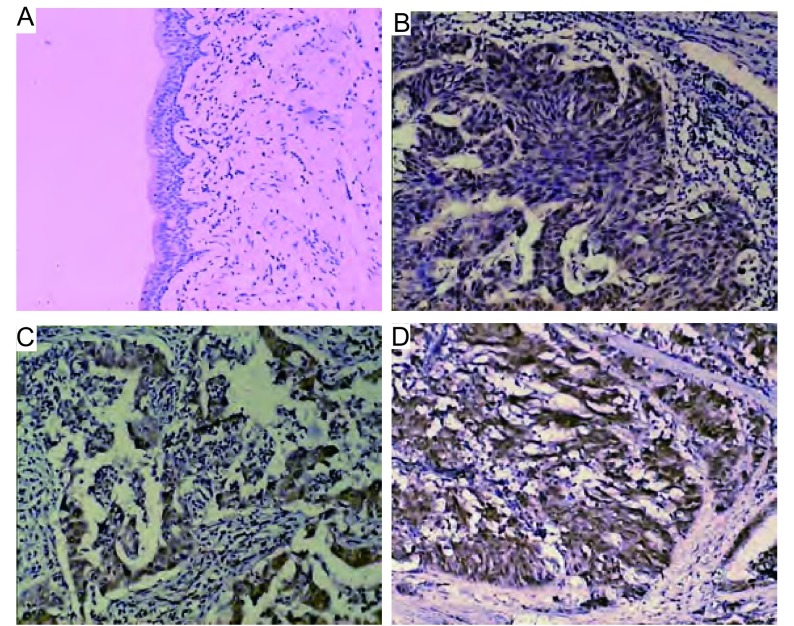
SHP2在肺癌组织中的表达(Invision法，×100)。A：SHP2在正常支气管粘膜中呈弱阳性；B、C和D：SHP2在肺鳞癌(B)、腺癌(C)和小细胞肺癌(D)中呈广泛强阳性。 SHP2 expression in lung cancer tissues (Invision, × 100). A: weak positive in normal bronchial epithelium; B, C and D: strong positive in squamous cell carcinoma (B), adenocarcinoma (C) and small cell lung cancer (D).

### 2.2 FISH检测结果

选择SHP2强阳性表达的病例进行FISH检测。与对照组相比，癌细胞胞质呈亮绿色，提示SHP2的mRNA有明显的扩增(图 2)，也进一步证实了免疫组化的结果。

**2:**
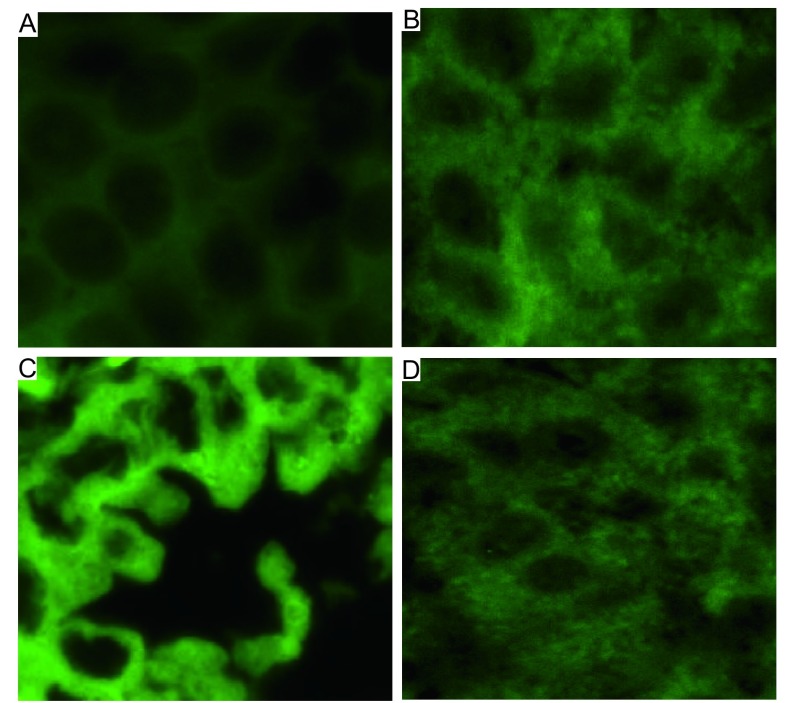
肺癌组织中SHP2 mRNA扩增(荧光原位杂交法，×400)。A：空白对照组：胞质呈淡绿色，为自发荧光；B、C和D：在鳞癌(B)、腺癌(C)及小细胞肺癌(D)中，胞质呈较亮的绿色，为扩增的SHP2 mRNA。 SHP2 mRNA amplification in lung cancer tissues (FISH, ×400). A: control group: dark green in the cytoplasm, which is autofluorescence; B, C and D: in squamous cell carcinoma (B), adenocarcinoma (C) and small cell lung cancer (D), bright green in the cytoplasm was seen, which is the amplified SHP2 mRNA.

### 2.3 SHP2的表达与肺癌组织学类型的关系

在15例正常支气管上皮中，SHP2大部分呈弱阳性(80%)；在肺癌组织中，SHP2约1/3为弱阳性(32.1%)，约1/2为中度阳性或强阳性(分别为47.2%和7.5%)，肺癌组织中SHP2的阳性率显著高于正常支气管上皮(*P*＜0.05)(表 1)。在48例NSCLC中，SHP2的弱阳性率为35.4%、中度阳性率为43.8%，强阳性率为6.2%；在5例SCLC中，SHP2的中度阳性率为80.0%，强阳性率为20.0%，SCLC中SHP2的阳性率显著高于NSCLC(*P*＜0.05)(表 2)。

**1:** SHP2在正常支气管上皮和肺癌组织中的表达 The expression of SHP2 in normal bronchial and lung cancer tissues

Group	*n*	Expression of SHP2 [*n* (%)]					*P*
		-	+	++	+++	Total	
Normal bronchial	15	3 (20.0%)	12 (80.0%)	0	0	12 (80.0%)	0.001
Lung cancer	53	7 (13.2%)	17 (32.1%)	25(47.2%)	4 (7.5%)	46 (86.8%)	

**2:** SHP2在NSCLC和SCLC中的表达 The expression of SHP2 in NSCLC and SCLC

Histological type	*n*	Expression of SHP2 [*n* (%)]					*P*
		-	+	++	+++	Total	
NSCLC	48	7 (14.6%)	17(35.4%)	21 (43.8%)	3 (6.2%)	41 (85.4%)	0.034
SCLC	5	0	0	4 (80.0%)	1 (20.0%)	5 (100%)	

### 2.4 SHP2的表达与吸烟指数的关系

在吸烟指数≥400患者的NSCLC癌组织中，SHP2的弱阳性率为23.8%，中度阳性率为71.4%，强阳性率为4.7%(总阳性率为100%)。在非吸烟NSCLC患者癌组织中，SHP2的弱阳性率为40.7%，中度阳性率为37.4%，强阳性率为3.7%(总阳性率为81.5%)，SHP2在吸烟指数≥400的癌组织中的阳性率高于非吸烟患者(*P*＜0.05)(表 3)。

**3:** 在NSCLC患者中SHP2表达与吸烟指数的关系 The relationship between SHP2 and smoking index among NSCLC patients

Smoking index	*n*	Expression of SHP2 [*n* (%)]					*P*
		-	+	++	+++	Total	
0	27	5 (18.5%)	11 (40.7%)	10 (37.4%)	1 (3.7%)	22 (81.5%)	0.011
≥400	21	0	5 (23.8%)	15 (71.4%)	1 (4.7%)	21 (100%)	
Among 5 cases of SCLC, 4 cases were recorded without smoking histories, so SCLC were not analyzed statistically.							

## 3 讨论

吸烟是肺癌最重要的危险因素之一，到2030年将有800多万人死于吸烟。烟草中所含的多环芳烃及亚硝胺类等致癌物可导致*Ras*、*p53*、*Rb*等基因点突变及*C*-*met*基因构成型激活等分子异常，进而导致肺癌的发生^[5]^。Ras-Raf-MAPK通路是吸烟导致肺癌的重要通路，*Ras*基因突变启动Ras-Raf-MAPK通路是吸烟导致肺癌的重要机制^[6]^，*KRAS*基因突变在大量吸烟者的癌组织中常见^[7]^。在本研究中，吸烟指数≥400的NSCLC癌组织中SHP2的阳性率高于非吸烟者，提示吸烟可能与SHP2表达增高相关。在吸烟NSCLC患者癌组织中，*RAS*基因突变与SHP2分子异常的关系如何？目前国内外未见报道，本课题组拟进一步开展相关研究。

SHP2是蛋白酪氨酸磷酸酶家族的成员之一，它在发育、生长、增殖、凋亡和分化等生物学过程中起重要作用^[8]^。SHP2在EGF、PDGF、FGF等触发的信号通路中起正性调节作用，在α-干扰素、γ-干扰素触发的Jak-Stat信号通路中起负性调节作用。SHP2可促进RAS的激活，其确切机制不明^[9]^。

研究^[10]^表明，SHP2是青少年粒-单核细胞性白血病、髓细胞性白血病、慢性粒-单核细胞性白血病的病因，在乳腺癌中表达率较高，与幽门螺旋杆菌相关胃癌的发生相关，有关SHP2在肺癌组织中的表达情况国内外文献较少。本研究表明，SHP2在肺癌组织中阳性率较高，最近国内的一个课题组也得出类似的结果^[11]^。这一结果提示，SHP2在肺癌的发生发展过程中可能发挥一定的作用，其具体机制有待深入研究。已知*EGFR*基因突变和*KRAS*基因突变是肺癌发病机制中最重要的两个突变^[7]^，可以通过检测肺癌组织中*EGFR*基因扩增或突变、*RAS*基因突变与SHP2的*PTPN11*基因扩增或突变之间的关系，从而确定在肺癌发病过程中SHP2是否及如何发挥重要作用。

目前，以蛋白酪氨酸激酶为靶点的药物研发已成为NSCLC治疗的研究热点之一，而SHP2作为蛋白酪氨酸磷酸酶的家族成员之一，在多种细胞信号通路中起着重要的调节作用^[12]^，其异常表达可导致多种疾病的发生，所以完全具备作为药物作用靶点开发的条件，本课题为这一方面的研究提供了新的依据。SCLC对放疗和化疗颇为敏感但很难治愈，而以SHP2为靶点研发新药为SCLC的药物治疗提供了新的思路。

磷酸化酪氨酸在NSCLC癌组织中的表达水平较高^[13]^，蛋白酪氨酸磷酸酶TEP1在肺癌组织中的表达远低于良性病变的肺组织^[14]^，而本研究中蛋白酪氨酸磷酸酶SHP2在肺癌组织中的表达显著增高。作者认为，肺癌组织中的磷酸化酪氨酸水平反应了整个酪氨酸磷酸酶的功能状态，可以通过进一步检测SHP2的磷酸酶活性与磷酸化酪氨酸水平之间的相关性以了解SHP2对肺癌组织中酪氨酸磷酸化水平的影响。
